# Removal of pathogens from greywater using green roofs combined with chlorination

**DOI:** 10.1007/s11356-022-23755-6

**Published:** 2022-10-27

**Authors:** Ioanna Petousi, Vasiliki Thomaidi, Nikolaos Kalogerakis, Michail S. Fountoulakis

**Affiliations:** 1 Department of Environment, University of the Aegean, Mytilene, Greece; 2 School of Chemical and Environmental Engineering, Technical University of Crete, Crete, Greece

**Keywords:** Wastewater, Regrowth, Green spaces, Water reuse, Disinfection, Coliforms

## Abstract

Greywater is an important alternative water resource which could be treated and reused in buildings, reducing the freshwater demand in drought affected areas. For the successful implementation of this solution, it is important to ensure the microbial safety of treated greywater. This study examined the microbiological quality of treated greywater produced by an emergent nature-based technology (green roofs) and a chlorination process. Specifically, the effect of substrate, substrate depth, and vegetation on the removal of total coliforms, *Escherichia coli*, and enterococci in experimental green roofs treating greywater was examined for a period of about 12 months. In addition, the ability of chlorination to inactivate the abovementioned pathogen indicators was evaluated and their potential regrowth was examined. Results shown that green roofs filled with 10 cm of perlite reduce total coliform concentration by about 0.4 log units while green roofs filled with 20 cm of vermiculite reduce total coliform concentration by about 1.2 log units. In addition, the use of vegetation in green roofs improves the removal of pathogenic bacteria by about 0.5 log units in comparison with unvegetated systems. In all cases, the effluents of green roofs failed to satisfy the criteria for indoor reuse of treated greywater for non-potable uses such as toilet flushing without a disinfection process. The addition of 3 mg/L of chlorine in the effluent provided safe greywater microbiological quality for storage periods of less than 24 h, while longer periods resulted in the significant regrowth of pathogens. In contrast, a chlorination dose of 7 mg/L completely secured inactivation of pathogen indicators for periods of up to 3 days.

**Supplementary Information:**

The online version contains supplementary material available at 10.1007/s11356-022-23755-6.

## Introduction

The treatment and utilization of greywater is a very interesting solution for promoting sustainable water management in cities (Lu et al. 2019). A significant volume of domestic wastewaters (45–60%) could be treated and reused on-site, reducing both the volume of freshwater demand in households (Humeau et al. 2011) and the energy required for the treatment of domestic wastewaters in centralized wastewater treatment plants (Friedler and Hadari 2006). Several treatment technologies have been tested in the past for greywater treatment including filtration (Katukiza et al. 2014), coagulation, rotating biological contactors (Abdel-Kader 2013), microalgae (Oktor and Çelik 2019), membrane bioreactors (MBRs), and wetlands. Among these, MBRs seem to achieve higher effluent quality (Chrispim and Nolasco 2017). However, their very high capital and operating costs, make the full scale application of MBRs in buildings challenging from an economic perspective (Fountoulakis et al. 2016). On the other hand, nature-based treatment systems (NBS), such as constructed wetlands, can achieve effluent quality similar to MBRs as far as physiochemical characteristics are concerned, while their operation costs are much lower. The disadvantage of these systems is that their installation requires significantly larger amounts of space, space that is not available in cities. Furthermore, constructed wetlands fail to satisfy the strict criteria for indoor reuse, demanding the addition of a disinfection unit (Arden and Ma 2018).

Recently, other types of NBS, which could be characterized as “modified wetlands,” such as green walls and green roofs, have been examined with respect to greywater treatment in an effort to minimize space requirements in buildings (Pradhan et al. 2019; Boano et al 2020). These types of NBS do not require extra space as they are installed on the unused outer surfaces of buildings. Previous findings have shown that green walls as well as green roofs could provide an effluent quality which meets the strict criteria for indoor reuse as regards physicochemical pollutants such as COD, BOD, turbidity, and suspended solids (Fowdar et al. 2017; Prodanovic et al. 2019). The type of porous media, in particular, as well as the type of vegetation used, seem important parameters for their ability to remove pollutants. For example, Thomaidi et al. (2022) found that vertical flow green roofs filled with vermiculite provide better effluent quality (regarding organic matter and solids) in comparison with vertical flow green roofs filled with perlite. Another study in India (Masi et al. 2016) showed that the use of coconut fibres instead of sand significantly improved COD removal in green walls treating greywater. Pradhan et al. (2020) examined six different materials — namely perlite, coco coir, lightweight expanded clay (LECA), date seeds, spent coffee grounds, and sand in vertical NBS for greywater treatment. They concluded that the use of sand, coco coir, and spent coffee grounds led to an increase in the removal of organic matter, solids, and nutrients in comparison with the other media examined. Nguyen et al. (2021) monitored a horizontal subsurface flow green roof for the post-treatment of domestic wastewater after a septic tank and reported that the removal of organic matter and nitrogen increased with the use of *Wedelia Trilobata* as green roof vegetation in comparison with *Axonopus Compressus.* In the same study, the use of charcoal as bed media resulted in significantly lower COD concentration in the effluents in comparison with the use of sand. It should be mentioned that lightweight materials, in general, are preferable for use in green roofs, in comparison with well-studied typical bed media such as sand and gravel, to keep the green roof weight below the structural limit of the building.

Knowledge about the fate of pathogens in green walls, and in green roofs in particular, is limited (Pradhan et al 2019). In general, several complex mechanisms related to porous media, vegetation, and hydraulic loading rate seem to affect pathogen removal in NBS (Wu et al. 2016). Ramprasad et al. (2017) examined the performance of a novel constructed wetland consisting of four rows of troughs filled with a mixture of sand, brickbat, and gravel for real greywater treatment and found a fecal coliform reduction of 2–3 log units. In contrast, Prodanovic et al. (2020) reported an *Escherichia coli* reduction of less than 1 log unit in an experimental green wall treating synthetic greywater. The medium used in this study was a mixture of perlite and coco coir at a ratio of 1:2. The same research team again found an *E. coli* and a total coliform reduction of about 1 log unit for a similar green wall system (Bakheet et al 2020). Recently, Lakho et al. (2021) operated a full-scale green wall in Belgium using a mixture of lava, organic soil and biochar (at a ratio of 2:1:1) as porous media and found a total coliform reduction of 2 log units.

In all cases, it is widely accepted that a disinfection unit must be installed in combination with a NBS for the safe reuse of treated greywater (Arden and Ma 2018). The disinfection technologies most used are chlorination, ozonation and ultraviolet radiation (UV). Among them, chlorination is the simplest and cheapest method for wastewater disinfection, making it ideal for the on-site treatment of greywater (Winward et al. 2008). Nevertheless, the major disadvantage of chlorination is the possible generation of toxic by-products such as chloramines, nitrosodimethylamine, and trihalomethane (Al-Gheethi et al. 2016). The quality of greywater as regards the presence of particles, organic compounds and nitrogen is important for an efficient chlorination process. For example, Mohamed et al. (2015) suggested doubled doses of chlorination for water with turbidity values greater than 100 NTU. Similar, Tal et al. (2011) found that the filtration of raw greywater and the addition of higher chlorination doses improves the inactivation of bacteria. Another important issue for safe greywater reuse is the potential regrowth of pathogenic bacteria during greywater storage. For this reason, it is suggested that the storage of the disinfected effluent for long periods of time is avoided (Boano et al. 2020). However, in practice, greywater could remain in storage tanks for periods longer than 2 days. The possible occurrence of organic compounds in the effluents not only increases the required chlorination doses, but also provides substrate for pathogenic bacteria regrowth (Winward et al 2008). Moreover, the presence of disinfectants in greywater resulted in increasing chlorine decay rates making it more difficult to control microbial regrowth (Tal et al. 2011). For these reasons, it is important to know the human health risk for any greywater treatment and reuse process. To date, the available data about the ability of green roofs to remove pathogen indicators from greywater is very limited. In addition, there is lack of knowledge regarding the kinetics of chlorination of green roof-treated greywater and potential regrowth of pathogenic bacteria.

In this context, this work examined the fate of several pathogen indicators (total coliforms, *Escherichia coli*, and enterococci) in green roofs treating greywater and the effect of porous media and vegetation on their removal. In addition, different chlorination doses and contact times were tested to define the appropriate chlorination scheme for safe storage and reuse for toilet flushing. These pathogen indicators were selected as they are the biological parameters defined in all existing guidelines and regulations for greywater reuse, worldwide (Arden and Ma 2018; Boano et al. 2020). However, it should be mentioned that other bacteria such as *Campylobacter jejuni*, *Pseudomonas aeruginosa*, and *Staphylococcus aerus* could be also very important for safe reuse as reported in several quantitative microbial risk analysis (Busgang et al. 2018; Shi et al. 2018).

## Material and methods

### Greywater

The recipe used for preparation of artificial light greywater was based on a well-known Australian protocol (Diaper et al 2008). Specifically, the ingredients added in 1000 L of tap water include shampoo (240 g), hand soap (240 g), toothpaste (21.7 g), moisturizing cream (6.7 g), deodorant (6.7 g), laundry (100 g), olive oil (4.7 g), urea (5 g), lactic acid (26.7), clay (33.3 g), and K_2_PO_4_ (2.6 g). In addition, to ensure the presence of pathogens, 10 L of primary-treated effluent obtained from the local sewage treatment plant of the University of the Aegean was added to the synthetic mixture. The characteristics of artificial greywater used in this study are presented in Table 1. The values recorded for both chemical and microbiological parameters were in accordance with those previously reported for real greywater (Fountoulakis et al. 2016; Boano et al. 2020).

**Table 1:** Chemical and microbiological characteristics of synthetic greywater used in the experiment and comparison with values reported in previous studies for real greywater

Parameter	Greywater Mean ± standard deviation (range)/number of samples	Literature^1^ Range
pH	8.0 ± 0.3 (7.3–8.4)/19	6.4–10
EC (mS/cm)	0.9 ± 0.2 (0.7–1.2)/19	0.6–1.6
Turbidity (FNU)	53 ± 28 (25–132)/17	37–173
COD (mg/L)	226 ± 60 (140–338)/16	26–645
BOD (mg/L)	132 ± 36 (85–210)/16	20–756
Total Coliforms (10^5^ MPN/100 mL)	15.1 ± 33.0 (0.1–95)/10	0.8–119
*E. coli* (10^5^ MPN/100 mL)	3.4 ± 6.3 (0.02–15)/10	0.01–49
Enterococci (10^5^ MPN/100 mL)	1.9 ± 1.3 (0.1–3.1)/8	0.01–5.1

^1^Fountoulakis et al. 2016;Boano et al. 2020

### Operation of green roofs

**Fig. 1:**
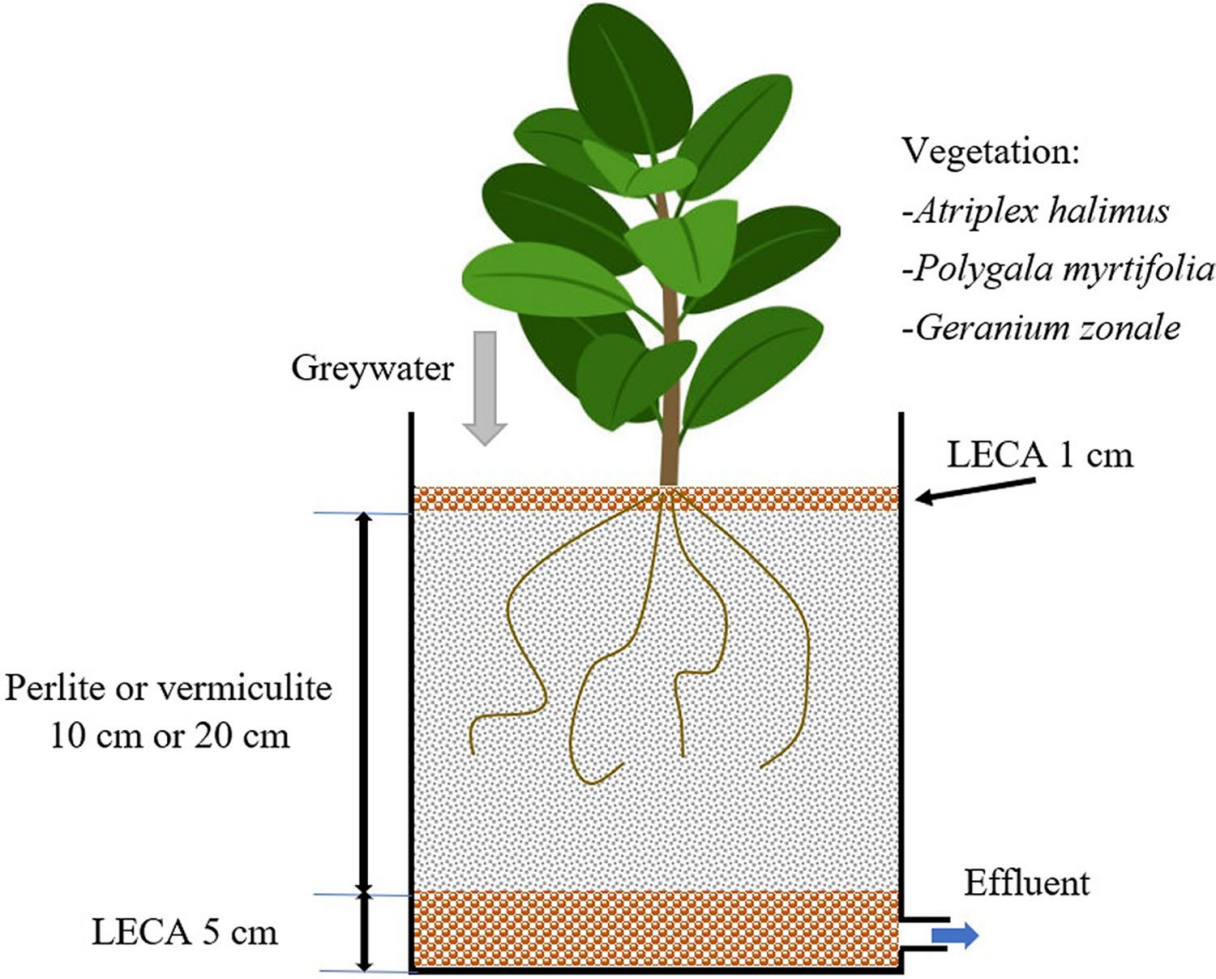
Schematic presentation of experimental green roofs

Detailed characteristics of the green roofs used in the experiment are described in a previous article (Thomaidi et al. 2022). Briefly, 80 plastic pots were installed on the roof of a building at the University of the Aegean (Fig. 1), in Mytilene, Greece, receiving artificial greywater for a period of about one year (from summer 2020 until summer 2021). Two different substrates (perlite, vermiculite), two different depths (10 cm and 20 cm), and four different types of vegetation (*Atriplex halimus*, *Geranium zonale*, *Polygala myrtifolia*, and no vegetation) were examined (Figure S1) while five replicates were used for each treatment (2 substrates × 2 depths × 4 plants × 5 replicates = 80 pots). Each pot was loaded four times per day with 0.8 L of greywater corresponding to a hydraulic loading rate (HLR) of about 45 mm/day. It is mentioned that VFCWs could achieve higher removal efficiencies in comparison with horizontal flow CW (per m^2^). For this reason, it was assumed that even shallow VFCWs may provide high-quality greywater effluents. In addition, an important technical issue for full scale applications is that the use of water saturated systems (such as horizontal flow CW) increases the green roof weight. This will require costly structural reinforcement for both existing and new buildings.

### Chlorination & regrowth

In a first step, sodium hypochlorite solution (10%) was added at several doses ranging from 1 to 7 mg/L in the effluent of green roofs to determine the chlorine demand. Two different qualities of green roof effluents were tested, namely “high quality” (from green roofs filled with 20 cm of vermiculite) and “low quality” (from green roofs filled with 10 cm of perlite). Residual chlorine was measured after 0.25 h, 0.5 h, and 1 h of contact time. The “high quality” effluent has a COD and an ammonium concentration of 10 mg/L and < 1 mg/L, respectively, while “low quality” effluent has a COD and an ammonium concentration of 142 mg/L and 1.2 mg/L, respectively.

In a second step, a “medium quality” green roof effluent containing 74 mg/L of COD and ammonium concentration < 1 mg/L was chlorinated with 3 mg/L, 5 mg/L, and 7 mg/L of chlorine. To determine regrowth potential, residual chlorine and biological indicators (total coliforms, *E. coli*) were monitored for a period of 72 h. All chlorination experiments were conducted in triplicate in Erlenmeyer flasks covered with aluminum foil (dark conditions) at room temperature (20 °C).

### Analytical methods

COD concentration of the samples was determined according to APHA (2005). The pH and the turbidity of raw and treated greywater was monitored with the use of a portable pH meter (C932, Consort) and turbidimeter (2100Q, Hach). Ammonium concentration and residual chlorine were measured spectrophotometrically using standard test kit (Hach). Samples from the influent and the effluent of experimental green roofs were collected every month (from September 2020 until April 2021) and analyzed for total coliforms, and *E. coli* by using Colilert-18 kit (IDEXX Laboratories Inc., USA). Briefly, greywater sample was poured into a Quanti-Tray, sealed in a Quanti-Tray sealer and placed in an incubator at 35 °C for 18 h. According to the manufacturer, wells with yellow color are positive for total coliforms and wells with blue fluorescence color are positive for *E. coli*. The most probable number (MPN) was determined from counted positive wells using the Quanti-Tray MPN Table provided by the manufacturer. In a similar procedure, enterococci were recorded by using Enterolert kits (IDEXX Laboratories Inc., USA). The incubation period for enterococci was 24 h at 41 °C. Sampling for all microbial analysis was carried monthly.

### Data analysis

The data were analyzed through two-way analysis of variance (ANOVA) to compare the effect of substrate type and substrate depth on effluent quality characteristics. Differences between means were determined by the Tukey test (Significance level: *p* < 0.05). Then, one-way ANOVA was used to determine significant differences in pathogens concentration for different substrates, depths, or vegetation. To meet the assumptions of ANOVA, all log_10_-transformed data were tested for normality and homogeneity of variance by Shapiro–Wilk and Levene’s test, respectively. All graphics and statistical tests were performed using OriginPro 2022 (Originlab, USA) software.

## Results and discussion

### Pollutants removal and plants growth

Details about the pollutant’s removal efficiency in experimental green roofs are presented in a previous article (Thomaidi et al. 2022). Briefly, higher removal efficiencies were observed for COD, TSS, and turbidity in green roofs filled with 20 cm of vermiculite ranged from 84 to 91%, 87 to 93%, and 85 to 93%, respectively. In contrast, the average removal of COD, TSS, and turbidity in green roofs filled with 10 cm of perlite was 39–45%, 44–53%, and 39–52%, respectively. A summary of the effluent quality in experimental green roofs during the operation are presented in Table 2. In general, the use of greater substrate depth and finer porous media have significant positive effect on the quality of treated greywater.

**Table 2:** Quality of treated greywater in experimental green roofs

Parameter	Substrate			
	Perlite		Vermiculite	
	10 cm	20 cm	10 cm	20 cm
pH	8.1 ± 0.2	8.2 ± 0.3	8.2 ± 0.2	8.3 ± 0.3
EC (mS/cm)	0.89 ± 0.14	0.87 ± 0.14	0.88 ± 0.14	0.86 ± 0.14
COD (mg/L)	131 ± 42	78 ± 38	64 ± 32	25 ± 17
BOD (mg/L)	80 ± 33	48 ± 28	40 ± 27	14 ± 10
TSS (mg/L)	19.8 ± 9.0	11.8 ± 5.7	9.2 ± 5.0	3.7 ± 3.2
Turbidity (FNU)	27.8 ± 13.4	15.5 ± 6.6	12.7 ± 6.4	5.3 ± 3.6

After almost 1 year, all plants of *A. halimus* and *G. zonale* were healthy without any visible symptoms of nutrient deficiency. In contrast, leaf discoloration and partial defoliation of *P. myrtifolia* plants was observed in some experimental green roofs mainly during the winter period. The average plant height of the plants at the end of experiment was 71.8 ± 7.9 cm, 47.5 ± 4.2 cm, and 22.8 ± 6.5 cm, for *A. halimus, P. myrtifolia* and *G. zonale*, respectively. It is mentioned that the weeds grown in the pots were removed by hand every month.

### Pathogen removal in green roofs

**Fig. 2:**
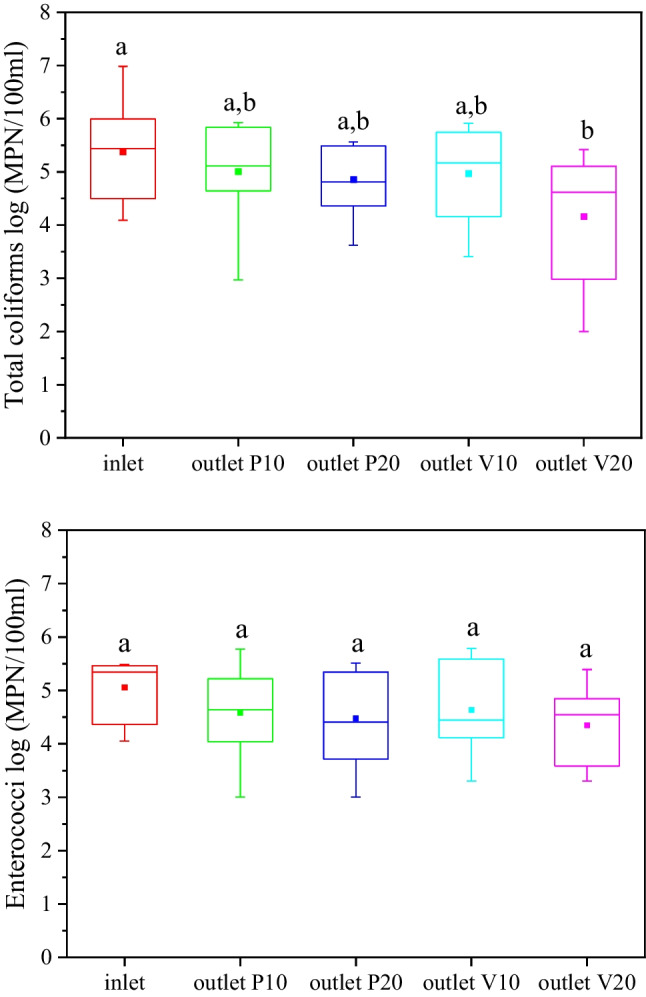
The presence of total coliforms (**a**) and enterococci (**b**) in the inlet and the outlets of vegetated green roofs during the experiment (number of samples: 12). Minimum and maximum values are indicated by the bottom and the top of the plot, respectively. Boxes represent median and lower and upper quartiles, while square points inside the box represent the mean values. P10: perlite 10 cm, P20: perlite 20 cm, V10: vermiculite 10 cm, V20: vermiculite 20 cm. Different letters indicate significant (*p* < 0.05) differences based on one-way ANOVA

Figure 2 shows the average removal of total coliforms and enterococci in green roofs throughout the experimental period. Mean total coliform reduction in the effluents of green roofs ranged from 0.4 log units in systems filled with 10 cm of perlite to 1.2 log units in systems filled with 20 cm of vermiculite. Similar, mean *E. coli* removal was found 0.4 log units, 0.7 log units, and 1.7 log units in systems filled with 10 cm of perlite, 20 cm of perlite or 10 cm of vermiculite and 20 cm of vermiculite, respectively. These values are lower than those previously recorded for vertical flow systems. Specifically, Kotsia et al. (2020) examined the removal of total coliforms in vertical flow constructed wetlands (VFCWs) filled with washed sand and found an average removal of 2.2 log units. Similarly, Arden and Ma (2018), in a review article, reported a total coliform reduction of 2.8 log units in VFCWs. Boano et al. (2020) report a total coliform reduction in pilot scale VFCWs treating greywater of about 3 log units. In the same article, they suggest that the main removal mechanisms of total coliform reduction are adsorption on substrate as well as the process of filtration. The filtration process has also been reported (Arias et al. 2001; Wu et al. 2016) as the main mechanism for the removal of several pathogen indicators (including total coliforms, fecal coliform, and fecal streptococci) in VFCWs treating domestic wastewater in the past. These suggestions explain the findings of this study too, as the substrate depths in the experimental green roofs were much lower (10–20 cm) than in typical VFCWs (50–80 cm). As a result, the adsorption and filtration process in green roofs is limited—resulting in lower removal rates. For these reasons, the mean total coliform concentrations in the effluents of green roofs filled with 20 cm of substrate are statistically significant (*p* < 0.05) lower in comparison with the effluents of green roofs filled with 10 cm of substrate (Table S1). Furthermore, green roofs filled with vermiculite had effluents with a slightly lower mean total coliform concentration in comparison with green roofs filled with perlite, due to the presence of finer particles (vermiculite 0.5–3 mm, perlite 1–5 mm), which enhance the filtration process. Prodanovic et al. (2018) examined *E. coli* removal in green walls filled with different mixtures of perlite and coir and concluded that physico-chemical processes are dominant in hydraulically faster mixes, while in slower mixes other mechanisms emerge, such as microbial degradation and predation. In this experiment, vermiculite was a hydraulically faster medium in comparison to perlite. As a result, enhanced total coliform removal could be also related to enhanced microbial degradation and predation processes.

The average enterococci reduction in experimental green roofs (Fig. 2b) was about 0.5 log units, while maximum mean reduction was recorded in the effluents of green roofs filled with 20 cm of vermiculite (0.7 log units). The scientific literature on the fate of enterococci in green roofs, as well as, in general, VFCWs is very limited. Winward et al. (2008) reported an enterococci removal of about 2 log units in a green roof water recycling system and a VFCW. The mechanisms related to enterococci removal in vertical flow systems are the same as the mechanisms related to total coliform removal. For this reason, the use of lower substrate depths and coarser particles resulted in a lower enterococci removal efficiency.

**Fig. 3:**
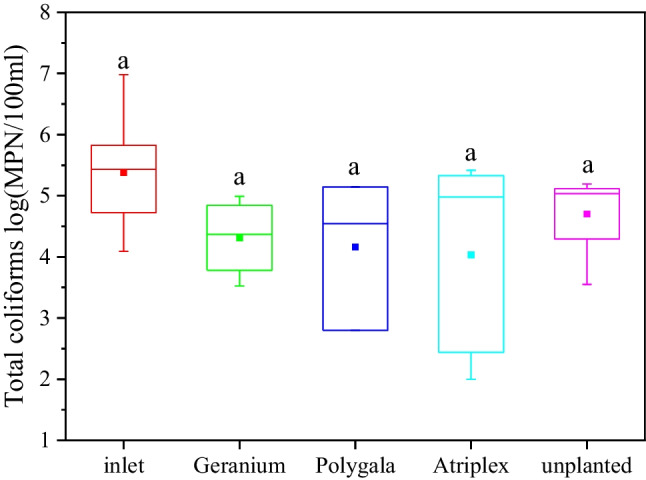
The effect of vegetation on total coliforms concentrations in the effluents of green roofs filled with 20 cm of vermiculite (number of samples: 12). Minimum and maximum values are indicated by the bottom and the top of the plot, respectively. Boxes represent median and lower and upper quartiles, while square points inside the box represent the mean values. Different letters indicate significant (*p* < 0.05) differences based on one-way ANOVA

The effect of vegetation on the removal of pathogen indicators is presented in Fig. 3. Mean total coliform concentration in the effluents of green roofs filled with 20 cm of vermiculite and planted with *A. halimus* was 1.3 log units lower in comparison to the influent, and 0.7 log units lower in comparison to the effluents, of the same green roofs when unplanted. Similar effluent quality was also recorded for the other two plants examined (*G. zonale* and *P. myrtifolia*). In general, it is known that the presence of plants in CWs treating domestic wastewater has a positive effect on pathogen reduction (Kadlec and Wallace 2009; Wu et al. 2016). Kotsia et al. (2020) examined the treatment of greywater in VFCWs planted with three different ornamental plants and found that vegetated systems reduce total coliforms and *E. coli* concentrations by about 0.3–0.6 log units more than unvegetated systems. The positive effect of vegetation on pathogen removal is related to the effect of plants on the hydraulic characteristics of the medium (Kadlec and Wallace 2009), as well as the increased surface area availability of plant roots (Kansiime and van Bruggen 2001), and the possible bactericidal activity of specific plants (Avelar et al 2014; Fountoulakis et al. 2017). For example, *A. halimus* is known for its extracts exhibiting antibacterial activity against several Gram-positive and Gram-negative pathogens (Abdel-Rahman et al. 2011). As a result, the exudates released by the plants could enhance the removal of pathogen indicators.

In any case, the effluent quality of all experimental green roofs failed to achieve the strict criteria for greywater reuse regarding microbiological characteristics. This observation is in accordance with previous results concerning the use of NBS (constructed wetlands, green roofs, green walls. etc.) for greywater treatment and reuse (Arden and Ma 2018; Boano et al. 2020). For this reason, an efficient disinfection unit must be added as post-treatment to meet reuse criteria.

### Chlorination

**Fig. 4:**
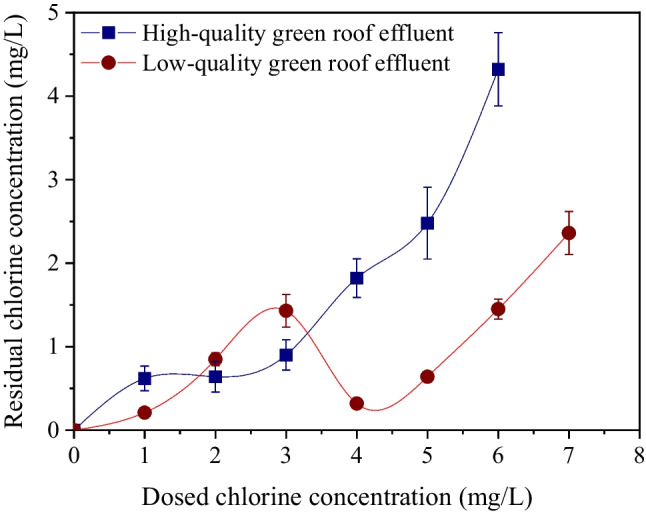
Chlorination curve for high-quality (COD:10 mg/L, NH_4_: < 1 mg/L) and low-quality (COD:142 mg/L, NH_4_: 1.2 mg/L) green roof effluents. Contact time: 30 min

Figure 4 presents the chlorination curves for two different qualities of green roof effluents. The “low-quality” effluent exhibits a break point at about 4 mg/L of dosed chlorine concentration. In contrast, the “high-quality” effluent exhibits a not well-defined break point at around 1.5 mg/L dose. It is known that the presence of ammonia and/or nitrogen-containing organic compounds in wastewater results in the production of chloramines, increasing the combined chlorine residual. Further chlorine doses result in a decline of combined chlorine residual to the point at which chlorine demand is satisfied and additional chlorine appears as free residual (breakpoint). The chlorine consumption after 30 min of contact time in both treatments of this experiment was somewhat lower than previously reported values for raw greywater. For example, Winward et al. (2008) report a chlorine consumption of about 10 mg/L while March and Gual (2007) report values more than 20 mg/L. The treatment of greywater with the use of green roofs resulted in the significant removal of organic matter, solids, and ammonium-nitrogen, reducing the chlorine demand in comparison with untreated greywater. Similar observations were also reported by Friedler et al. (2011) in a previous work where they examined the chlorination of greywater treated by a rotating biological contactor and a sedimentation basin.

**Fig. 5:**
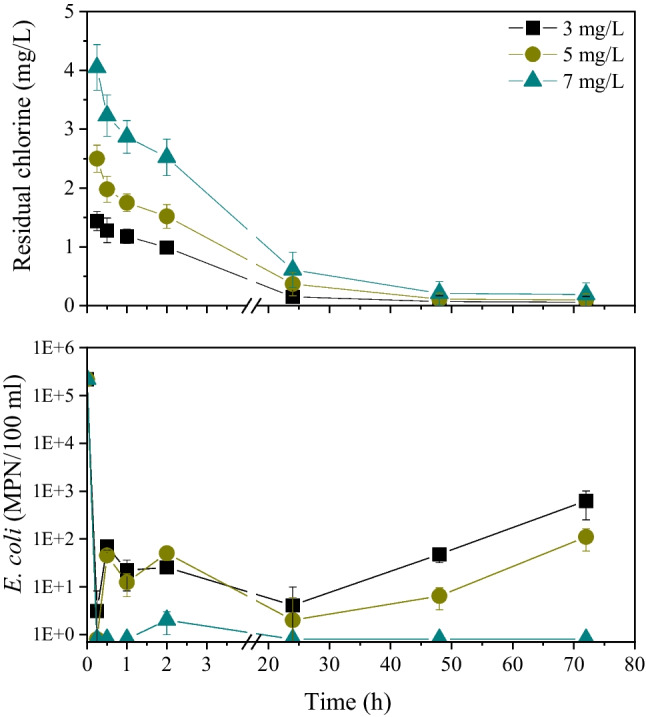
Residual chlorine concentration and *E. coli* inactivation and regrowth during the experiment with a medium-quality effluent (COD: 74 mg/L, NH_4_: < 1 mg/L)

Figure 5 shows the inactivation of *E. coli* in the effluent of green roofs at three different chlorination doses (3, 5 and 7 mg/L). *E. coli* concentration decreased from 22,000 ± 1251 MPN/100 mL before chlorination to 69.7 ± 11.1 MPN/100 mL, 45.3 ± 8.2 MPN/100 mL and < 1 MPN/100 mL after 0.5 h of chlorination with 3 mg/L, 5 mg/L, and 7 mg/L of chlorine, respectively. The inactivation of *E. coli* continued for a period of about 24 h when the *E. coli* concentrations reach minimum values of 4.1 MPN/100 mL, 2 MPN/100 mL, and < 1 MPN/100 mL in the treated greywater dosed with 3 mg/L, 5 mg/L, and 7 mg/L of chlorine, respectively. During the next two days, *E. coli* concentration increased at 1–2 orders of magnitude in the effluent chlorinated with 3 mg/L and 5 mg/L of chlorine. In contrast, the green roof effluent dosed with 7 mg/L of chlorine remained free of *E. coli* even 3 days after chlorination.

Similar behavior was also observed for total coliform inactivation. Specifically, total coliform concentration decreased from 600,000 ± 4500 MPN/100 mL to 200.5 ± 24.3 MPN/100 mL, 94.5 ± 38.7 MPN/100 mL and < 1 MPN/100 mL after 0.5 h of chlorination with 3 mg/L, 5 mg/L and 7 mg/L of chlorine, respectively. Three days after chlorination total coliform concentration increased to values up to 1.8 × 10^7^ in the effluents dosed with 3 mg/L of chlorine. On the other hand, the effluents dosed with 7 mg/L of chlorine had a total coliform concentration of about 100 MPN/100 ml.

Pathogen indicator inactivation is clearly related to the residual chlorine concentration in the effluents (Fig. 5a). The use of 7 mg/L of chlorine resulted in significantly higher residual chlorine concentration throughout the experiment, in comparison with other dosing schemes. In addition, the chlorine consumption rate was higher during the first minutes of chlorination (2–4 mg/L), while from 0.5 h to the end of the experiment the chlorine decay rate decreased significantly. It seems that even after 24 h of chlorination the residual chlorine is enough to prevent regrowth of pathogen indicators in the storage tank of treated greywater. Specifically, the residual chlorine after 24 h of chlorination was found to be 0.15 mg/L, 0.37 mg/L, and 0.61 mg/L in flasks dosed with 3 mg/L, 5 mg/L, and 7 mg/L of chlorine, respectively. On the other hand, the retention of treated greywater in tanks for periods longer than 24 h led to the regrowth of pathogens, increasing the risk to human health.

Friedler et al. (2011) examined the chlorination of rotating biological contactor-treated greywater and found that effluents with residual chlorine of 0.5 and 1.0 mg/L not only inactivated bacteria regrowth, but also resulted in further gradual inactivation for a period of 6 h, similar with this study. In addition, Rose et al. (1991) reported that coliforms increased to 1 and 2 log CFU/100 mL during the storage period of over 48 h, in accordance with our findings. In summary, the microbiological characteristics of green roof effluents obtained in this study after chlorination are presented and compared with worldwide guidelines given in Table 3. *E. coli* concentration during different chlorination doses and storage periods ranged from < 1 to 630 MPN/100 mL while criteria for greywater reuse in toilets ranged from < 1 MPN/100 mL in Australia to < 100 MPN/100 mL in Israel and the USA. The addition of 7 mg/L of chlorine could meet the strict criteria for indoor reuse even after 3 days of storage. In contrast, the use of lower chlorination doses requires very short storage periods for safe greywater reuse. It has been said that the reuse of greywater for toilet flushing minimizes the risk from possible presence of toxic chlorination by-products in the chlorinated effluents, as the treated greywater ends up at sewage treatment plants. There, these toxic compounds are treated by aerobic bacteria and other wastewater treatment processes (sedimentation, filtration, anaerobic digestion etc.) increasing the possibility of their elimination prior to disposal in the environment (Rostad et al. 2000; Chen et al. 2014).

**Table 3:** *E. coli* concentration (MPN/100 ml) achieved during this study in comparison with guidelines and regulations for greywater reuse for toilet flushing

This study				Guidelines	
Storage period	Dose				
	7 mg/L	5 mg/L	3 mg/L	UK	< 25
24 h	< 1	4.1	2.0	Israel	< 100
48 h	< 1	6.4	47.8	USA	< 100
7 2 h	< 1	110	630	Australia	< 1

^1^British standards Institute BS8525-2 (2011)

^2^California Title 22 as reported byYu et al. (2013)

^3^SI-6147 as reported by Oron et al. (2014)

^4^Goverment of Western Australia (2011)

## Conclusions

The goal of this work was to examine the effect of green roof design on pathogen removal from greywater. According to the findings, the increase of substrate depth resulted in an increase in pathogen removal. The filtration process seems to be the dominant removal mechanism of pathogenic bacteria in all NBS. In addition, vegetated systems provided better effluent quality regarding pathogenic bacteria concentration in comparison with unvegetated systems probably due to the release of exudates. In all cases, low removal efficiencies of pathogen indicators were recorded ranged from 0.4 to 1.2 log units. For this reason, chlorination is a necessary step for the safe reuse of treated greywater. The presence of higher amounts of organic matter in the effluents of green roofs resulted in a higher chlorine demand. The addition of chlorination doses ranging from 3 to 7 mg/L produced chlorinated effluents which met microbiological criteria for indoor reuse if the storage periods did not exceed 24 h. For longer storage periods a chlorination dose of 7 mg/L should be chosen to ensure safe greywater reuse for toilet flushing. Overall, the combination of a green roof with a simple post-chlorination process could efficiently treat the greywater, providing an appropriate effluent quality for indoor non-potable uses.

## Supplementary Information

Below is the link to the electronic supplementary material.

Supplementary file1 (DOCX 1545 KB)

## Data Availability

Not Applicable.
