# Supplementary material for: Removal of pathogens from greywater using green roofs combined with chlorination
Source: Environ Sci Pollut Res Int. 2022 Oct 27;30(9):22560–9. doi: 10.1007/s11356-022-23755-6 (PMC9938822; doi:10.1007/s11356-022-23755-6)
Supplement: Supplementary file 1 — Supplementary file1 (DOCX 1545 KB) [file 11356_2022_23755_MOESM1_ESM.docx]

**Removal of pathogens from greywater using green roofs combined with chlorination**

Supplementary data


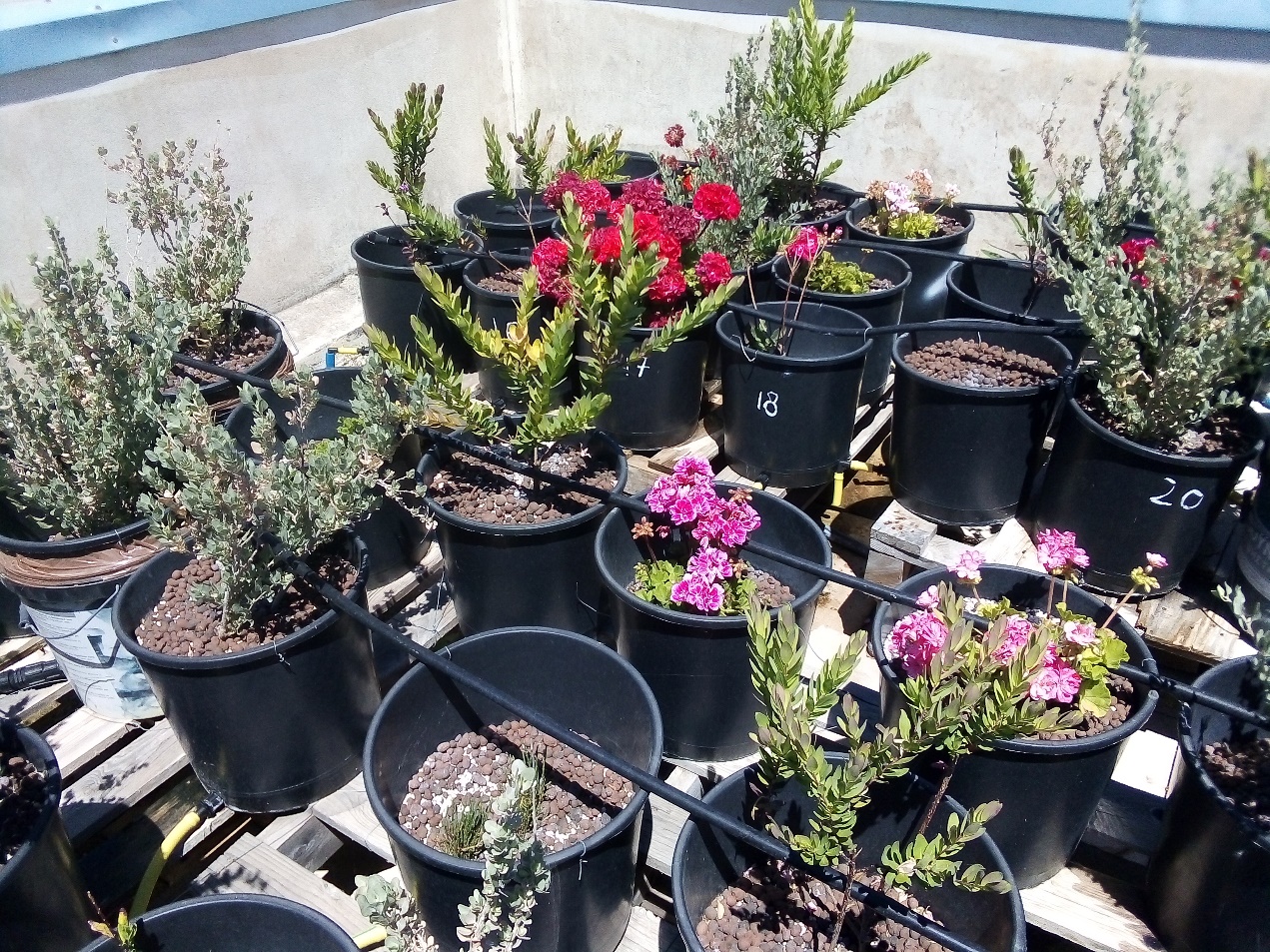


*Geranium*

*zonale*

*Atriplex halimus*

*Polygala*

*myrtifolia*

*Unvegetated*


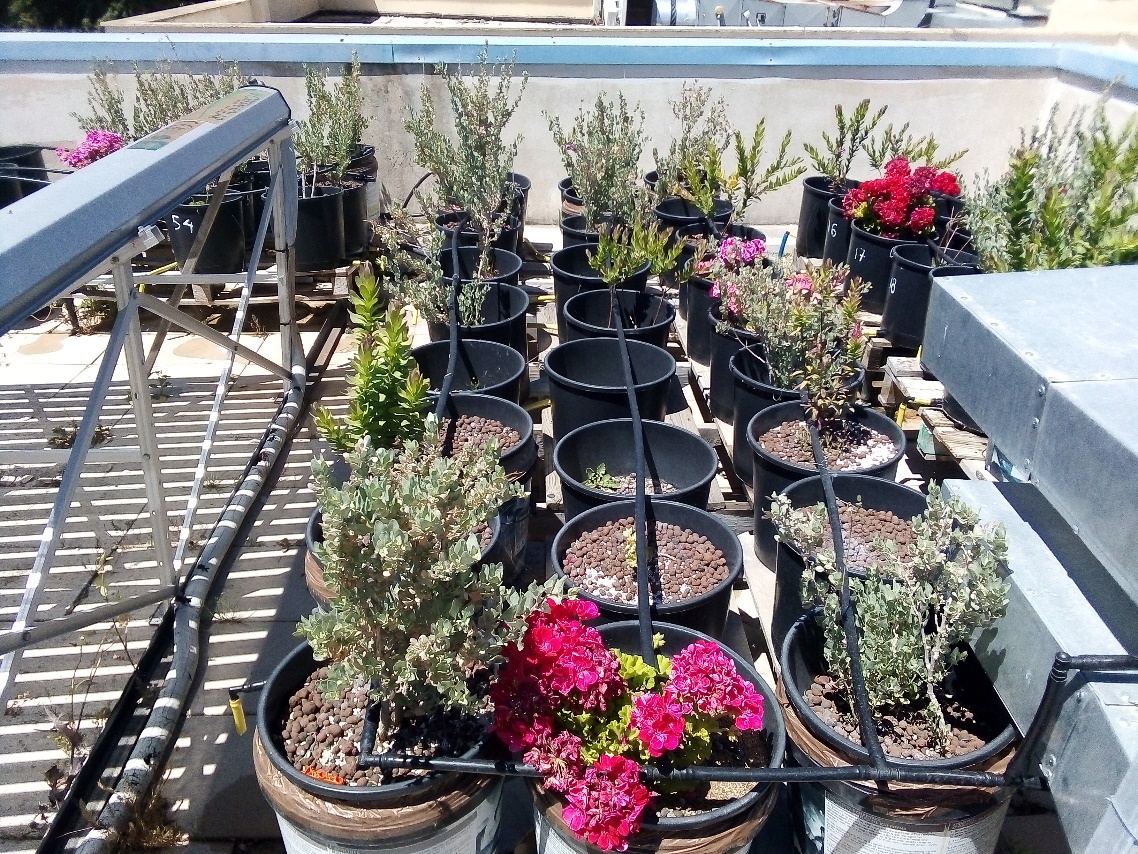


**Figure S1.** View of experimental green roofs (Spring 2021)


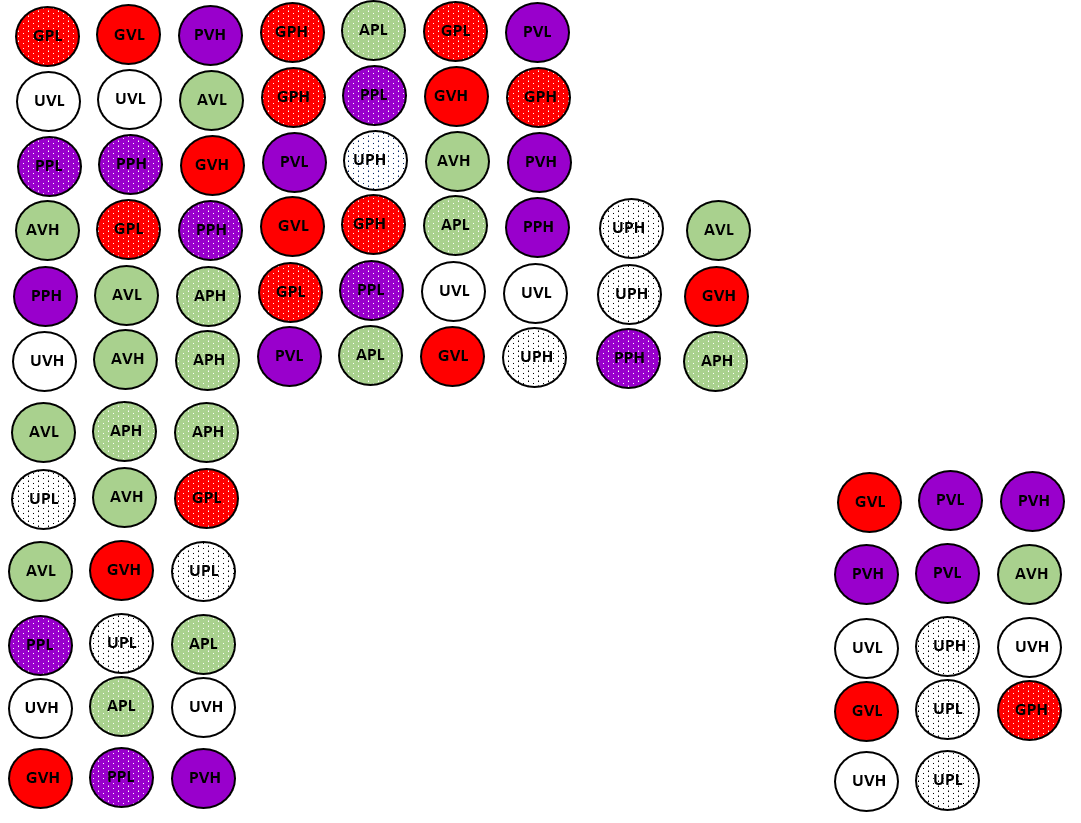


**Figure S2.** Experimental design layout of green roofs. First letter: G: *Geranium zonale*, A: *Atriplex halimus*, P: *Polygala myrtifolia*, U: unplanted, Second Letter: P: perlite, V: Vermiculite, Third letter: L: 10 cm, H: 20 cm

**Table S1.** Two-way ANOVA and post hoc Tukey test regarding total coliforms concentration in the effluents of experimental green roofs

Overall

|  | DF | Sum of squares | Mean square | F-value | p-value |
| --- | --- | --- | --- | --- | --- |
| Substrate | 1 | 2.43025 | 2.43025 | 3.06424 | 0.08734 |
| Depth | 1 | 3.80572 | 3.80572 | 4.79853 | **0.03409** |
| Interaction | 1 | 0.64168 | 0.64168 | 0.80907 | 0.37352 |

Means comparison

Substrate

| Substrate | meanDiff | SEM | q-value | Prob |
| --- | --- | --- | --- | --- |
| Vermiculite-perlite | -0.46014 | 0.26286 | 2.47558 | 0.08734 |

Depth

| Depth | meanDiff | SEM | q-value | | Prob |
| --- | --- | --- | --- | --- | --- |
| 20 cm - 10 cm | -0.58609 | 0.26261 | 3.1562 | **0.03102** | |

Interactions

| Substrate | Depth | Substrate | Depth | meanDiff | SEM | q-value | Prob |
| --- | --- | --- | --- | --- | --- | --- | --- |
| perlite | 20 cm | perlite | 10 cm | -0.33937 | 0.37974 | 1.26389 | 0.80813 |
| vermiculite | 10 cm | perlite | 10 cm | -0.2237 | 0.37174 | 0.85101 | 0.93094 |
| vermiculite | 10 cm | perlite | 20 cm | 0.11567 | 0.37174 | 0.44006 | 0.9894 |
| vermiculite | 20 cm | perlite | 10 cm | -1.03595 | 0.37174 | 3.94105 | **0.03833** |
| vermiculite | 20 cm | perlite | 20 cm | -0.69658 | 0.37174 | 2.64998 | 0.25464 |
| vermiculite | 20 cm | vermiculite | 10 cm | -0.81225 | 0.36357 | 3.15949 | 0.13069 |

**Table S2.** Two-way ANOVA and post hoc Tukey test regarding enterococci concentration in the effluents of experimental green roofs

**Overall**

|  | DF | Sum of squares | Mean square | F-value | p-value |
| --- | --- | --- | --- | --- | --- |
| Substrate | 1 | 0.01133 | 0.01133 | 0.01676 | 0.89776 |
| Depth | 1 | 0.35442 | 0.35442 | 0.52436 | 0.47394 |
| Interaction | 1 | 0.08233 | 0.08233 | 0.12181 | 0.72924 |

**Means comparison**

Substrate

| Substrate | meanDiff | SEM | q-value | Prob |
| --- | --- | --- | --- | --- |
| Vermiculite-perlite | -0.03968 | 0.26711 | 0.2101 | 0.88278 |

Depth

| Depth | meanDiff | SEM | q-value | Prob |
| --- | --- | --- | --- | --- |
| 20 cm-10 cm | -0.19094 | 0.26711 | 1.01096 | 0.47958 |

Interactions

| Substrate | Depth | Substrate | Depth | meanDiff | SEM | q-value | Prob |
| --- | --- | --- | --- | --- | --- | --- | --- |
| perlite | 20 cm | perlite | 10 cm | -0.10044 | 0.36952 | 0.38438 | 0.99285 |
| vermiculite | 10 cm | perlite | 10 cm | 0.05879 | 0.36952 | 0.22498 | 0.99854 |
| vermiculite | 10 cm | perlite | 20 cm | 0.15922 | 0.38756 | 0.581 | 0.97623 |
| vermiculite | 20 cm | perlite | 10 cm | -0.22854 | 0.36952 | 0.87466 | 0.92547 |
| vermiculite | 20 cm | perlite | 20 cm | -0.12811 | 0.38756 | 0.46746 | 0.98732 |
| vermiculite | 20 cm | vermiculite | 10 cm | -0.28733 | 0.38756 | 1.04846 | 0.87966 |
